# Acquisition of Type I methyltransferase via horizontal gene transfer increases the drug resistance of *Aeromonas veronii*

**DOI:** 10.1099/mgen.0.001107

**Published:** 2023-09-27

**Authors:** Jiayue Ma, Honghao Zhao, Shuangyi Mo, Juanjuan Li, Xiang Ma, Yanqiong Tang, Hong Li, Zhu Liu

**Affiliations:** ^1^​ School of Life Sciences, Hainan University, Haikou, PR China

**Keywords:** *Aeromonas veronii*, drug resistance, horizontal gene transfer, methylome, Type I restriction-modification system

## Abstract

*
Aeromonas veronii* is an opportunistic pathogen that affects both fish and mammals, including humans, leading to bacteraemia, sepsis, meningitis and even death. The increasing virulence and drug resistance of *A. veronii* are of significant concern and pose a severe risk to public safety. The Type I restriction-modification (RM) system, which functions as a bacterial defence mechanism, can influence gene expression through DNA methylation. However, little research has been conducted to explore its origin, evolutionary path, and relationship to virulence and drug resistance in *A. veronii *. In this study, we analysed the pan-genome of 233 *A *. *veronii* strains, and the results indicated that it was 'open', meaning that *A. veronii* has acquired additional genes from other species. This suggested that *A. veronii* had the potential to adapt and evolve rapidly, which might have contributed to its drug resistance. One Type I methyltransferase (MTase) and two complete Type I RM systems were identified, namely AveC4I, AveC4II and AveC4III in *A. veronii* strain C4, respectively. Notably, AveC4I was exclusive to *A. veronii* C4. Phylogenetic analysis revealed that AveC4I was derived from horizontal gene transfer from *Thiocystis violascens* and exchanged genes with the human pathogen *Comamonas kerstersii *. Single molecule real-time sequencing was applied to identify the motif methylated by AveC4I, which was unique and not recognized by any reported MTases in the REBASE database. We also annotated the functions and pathways of the genes containing the motif, revealing that AveC4I may control drug resistance in *A. veronii* C4. Our findings provide new insight on the mechanisms underlying drug resistance in pathogenic bacteria. By identifying the specific genes and pathways affected by AveC4I, this study may aid in the development of new therapeutic approaches to combat *A. veronii* infections.

## Data Summary

The authors confirm all supporting data have been provided within the article or through supplementary data files.

**Impact Statement**

Numerous studies have reported that methylation of the Type I restriction-modification (RM) system affects gene expression regulation, virulence modulation and drug resistance in several pathogens. In recent years, *Aeromonas veronii* has become a focus of research due to its growing drug resistance, which poses a notable threat to human health and the aquatic economy. However, despite its significance, the function and role of the Type I RM system in *A. veronii* continue to remain elusive. In this investigation, we sequenced the genome of *A. veronii* C4 and conducted comparative genomic analysis with 19 other *A. veronii* strains in the database. Our analysis indicated that *A. veronii* possesses an open pan-genome, and *A. veronii* C4 displayed a distinctive Type I RM system methyltransferase gene in comparison to other strains. Horizontal gene transfer predictions in bacteria and archaea indicated that the methyltransferase gene might have been acquired from *Thiocystis violascens* and *Comamonas kerstersii *. The gene was found to be associated with drug resistance by methylation analysis. Overall, our findings suggest that the epigenetic changes caused by the horizontally acquired methyltransferase gene of the Type I RM system considerably contribute to enhanced drug resistance in *A. veronii *.

## Introduction

Initially considered to be part of the innate immune system in bacteria, recent studies have shown that restriction-modification (RM) systems can also modify specific DNA sites through methylation, shaping bacterial epigenomes and regulating traits such as virulence and adaptability [1, 2]. RM systems are divided into four types (I, II, III and IV) [3]. The Type I RM system is the most intricate, encoded by three *hsd* (host specificity of DNA) genes, namely *hsdR*, *hsdM* and *hsdS* [4]. The genes *hsdM* and *hsdS* are closely located under the control of the same promoter, while *hsdS* is unique to the Type I RM system [5]. A restriction endonuclease (R_2_M_2_S_1_) is composed of two restriction (R) subunits encoded by *hsdR*, two modification (M) subunits encoded by *hsdM* and one specificity (S) subunit encoded by *hsdS*. The M_2_S_1_ complex of the R_2_M_2_S_1_ endonuclease solely functions as a DNA methyltransferase (MTase) [6]. The Type I RM system cleaves nonmethylated foreign DNA using R_2_M_2_S_1_, while protecting endogenous DNA through M_2_S_1_ methylation [4, 7, 8]. The S subunit is shaped like a dumbbell and contains a simple sequence repeat (SSR) in the centre, as well as various target recognition domains (TRDs) at both ends [6]. The length of the SSR limits the number of nonspecific bases ‘N’ in the motif, while the TRD determines the specific DNA sequence [9]. Apart from the classical Type I RM system mentioned above, which generates 6mA modifications, the noncanonical system utilizes two different M subunits to form MTase instead of identical copies, modifying m6A on one strand and m4C on the other [10–12].

The Type I RM system possesses the characteristics of mobile genetic elements (MGEs) and facilitates gene exchange between strains possessing homologous Type I RM systems. Theoretically, the presence of a greater number of Type I RM systems within a strain is positively associated with an increased gene abundance and frequency of gene exchange [13]. Furthermore, as observed in various pathogens such as *Neisseria meningitidis* [14], *Pseudomonas aeruginosa* [15], *Staphylococcus aureus* [16], certain species of the genus *Streptococcus* [17–19], *Mycoplasma* [20, 21] and others [22–25], the Type I RM system regulates gene expression through methylation modification, which subsequently affects strain phenotypes. Inactivation of the MTase of *Pseudomonas aeruginosa* reveals an attenuated-virulence phenotype in the *Galleria mellonella* infection model [15]. Variability in the repeat sequences of the Type I RM system of *Mycoplasma pneumoniae* determines its macrolide resistance [21]. However, most recent studies have focused on the funtion of the Type I RM system, and its origin and evolutionary traits have yet to be traced.

*Aeromonas veronii* is a rod-shaped, Gram-negative, facultatively anaerobic bacterium with strong motility, virulence and environmental adaptability, making it ubiquitous worldwide [26, 27]. It is primarily isolated from fish lesions and causes symptoms such as haemorrhages, ulceration and visceral enlargement, resulting in significant financial losses in the aquaculture industry [28]. Additionally, it infects amphibians, reptiles and mammals, and spreads through wounds, leading to a variety of diseases and even death [29]. Of particular concern is the increasing virulence and drug resistance of *A. veronii *, which pose a significant threat to public health [27, 30–35]. Bacterial whole-genome analysis serves as an essential method for understanding the genetic variations, functionality and evolution of bacteria, offering basic support and evidence for disease prevention and environmental conservation [36, 37]. Although the genomes of individual *A. veronii* strains have been sequenced, little is known about the key regulatory elements such as the Type I RM systems and their contribution to pathogenesis and drug resistance. For *A. veronii *, analysis of its complete genome, specifically elucidating the presence and distribution patterns of the Type I RM system, bears substantial significance for the prevention and treatment of infections caused by this pathogen. It remains unclear whether *A. veronii* acquired the Type I RM system from other bacteria during its evolution, or whether there was horizontal transfer of this system between *A. veronii* and other bacterial species.

The present study employed genomic sequencing and comparative analysis to investigate *A. veronii* strain C4. In addition, the epigenome of *A. veronii* C4 was examined to identify the specific motif methylated by AveC4I. Subsequently, the obtained data were subject to functional annotation and enrichment analysis. This work holds theoretical and practical value in elucidating the drug-resistant mechanisms of the pathogen, identifying novel drug targets, and exploring new strategies for pathogen control.

## Methods

### Genome sequencing and assembly

In 2015, *A. veronii* C4 was isolated from the liver lesions of diseased *Ctenopharyngodon idella* in Shantou, China [38, 39]. Since then, the strain has been stored in our laboratory. Single colonies were picked and cultured in LB liquid medium at 30 °C with shaking at 150 r.p.m. for 16 h. The bacterial cells were then collected and stored at −80 °C until further analysis. Genomic DNA was extracted using the MgiEasy Microbial DNA Extraction Kit.

For Illumina sequencing, DNA fragments were generated using an ultrasonic DNA breaker (Covaris), followed by end-repair, 3′-adenylation, and ligation to the adaptors. The amplified and purified fragments were used to construct high-quality library, which was sequenced on the Illumina HiSeq 4000 platform as paired-end 2×150 bp reads.

For single molecule real-time (SMRT) sequencing, DNA fragments of 10–15 kb were treated with enzymatic digestion, damage-repair and end-repair, and connected with the barcode sequence linker. The SMRTbell library was obtained and sequenced on the PacBio RS II platform. Both Illumina and SMRT sequencing were performed at Beijing Genomics Institute (BGI).

To ensure the accuracy of the results, Illumina and SMRT reads with low-quality (≤20) bases (40 % as default) or Ns exceeding a certain threshold (10 % as default), adapter and duplication contamination, and SMRT reads shorter than 1000 bp were removed. The SMRT reads were self-corrected and assembled using Pbdagcon (https://github.com/PacificBiosciences/pbdagcon) and Celera Assembler (http://wgs-assembler.sourceforge.net), respectively. The assembled genome was corrected by GATK (https://www.broadinstitute.org/gatk/) and SOAP tools (including SOAP2, SOAPsnp and SOAPindel) based on Illumina reads. The complete genome of *A. veronii* C4 was submitted to the NCBI GenBank database (accession number CP110364). Gene prediction was performed using glimmer3 (http://ccb.jhu.edu/software/glimmer/index.shtml) with the Hidden Markov model. tRNAs, rRNAs and sRNAs were identified by comparing with the tRNAscan-SE [40], RNAmmer [41] and Rfam [42] databases. Protein sequence alignments against the Non-Redundant Protein Sequence (NR), UniProt, Kyoto Encyclopedia of Genes and Genomes (KEGG) and Pfam databases were carried out to obtain *hsd* genes in *A. veronii* C4

### Pan-genome analysis

The genomes of 19 other *A. veronii* strains were obtained from the NCBI Genome database using the NCBI Datasets command-line tool with the parameters ‘datasets download genome taxon “aeromonas veronii” --assembly-level complete_genome --assembly-source refseq --dehydrated’ in November 2021. RefSeq assembly accession numbers are provided as follows: GCF_000204115.1 [43], GCF_000464515.2 [44], GCF_001593245.1, GCF_002803925.1, GCF_002803945.1, GCF_003491365.1, GCF_003722175.1, GCF_008693705.1 [45], GCF_009755745.1, GCF_009834065.1, GCF_011045495.1, GCF_013415825.1, GCF_014168715.1 [46], GCF_014168995.1 [46], GCF_014169795.1 [46], GCF_014169835.1 [46], GCF_014169875.1 [46], GCF_017310275.1 and GCF_020172765.2 [47]. To ensure the timeliness and comprehensiveness of the data, we also removed the restriction of assembly level and downloaded 232 *A *. *veronii* genomes on 28 July 2023. Pan-genome analysis was performed using Bacterial Pan Genome Analysis (BPGA) [48]. The results were visualized with gnuplot software and the Tutools platform (http://www.cloudtutu.com). OrthoFinder was used to identify homologous genes of the Type I RM systems in *A. veronii* C4 [49]. The Wagner parsimony method implemented in the Count software package was used to infer the orthologous genes of ancestors, with a gain/loss penalty rate of 2 [50].

### Phylogenetic analyses of HsdM1, HsdS1 and *Aeromonas* species

The protein annotations of fully assembled reference genomes of 3357 bacteria and 220 archaea were downloaded from the NCBI Genome database using the NCBI Datasets command-line tool with the parameters ‘datasets download genome taxon bacteria --assembly-level complete_genome --reference --dehydrated’ and ‘datasets download genome taxon archaea --assembly-level complete_genome --reference --dehydrated’. Homologous proteins of HsdM1 and HsdS1 in *A. veronii* C4 were identified by aligning their sequences against HsdM1 and HsdS1 using a sequence identity threshold of 30 % and coverage threshold of 40 %. The program muscle was used to perform multiple sequence alignments [51], and IQ-TREE was executed to reconstruct phylogenetic trees of HsdM1 and HsdS1 based on the maximum likelihood method [52]. Species that contained homologous HsdM1 and HsdS1 were selected, and single-copy core genes from these species were identified using OrthoFinder. Multiple sequence alignments for each orthogroup were carried out using muscle, and spurious sequences or poorly aligned regions were removed using trimAL [53]. High-quality-alignment single-copy core genes were concatenated, and further were used to reconstruct the phylogenetic tree of the species containing homologous HsdM1 or HsdS1 by IQ-TREE. The taxonomy of the species was annotated using Taxonkit [54]. All phylogenetic trees were visualized using iTOL [55]. Gene clusters were visualized using ChiPlot (https://www.chiplot.online/). The reliability of the phylogenetic trees was evaluated using 1000 bootstraps.

### Inference of evolutionary relationships

The phylogenetic tree of species containing homologous HsdM1 or HsdS1, as well as the phylogenetic trees of HsdM1 or HsdS1 were analysed using Ranger-DTL to infer gene evolution [56]. In total, 1000 reconciliations were calculated for each gene with different random seeds, and the results were aggregated using the AggregateRanger function. Gene duplication or horizontal gene transfer (HGT) events were considered supported if the corresponding support value was at least 50 %.

### Methylation analysis of the SMRT sequencing data

The genome index of *A. veronii* C4 was generated using samtools, and the SMRT reads were aligned to the genome using pbalign. The software ipdSummary was then utilized to identify 6mA sites, and the methylated motifs containing the 6mA sites were detected using motifMaker. A motif was defined as a DNA sequence pattern that occurred repeatedly. These steps were carried out in the SMRT Analysis platform (v2.3.0, http://www.pacb.com/products-and-services/analyticalsoftware/smrt-analysis/analysis-applications/epigenetics/). The Blast tool in the REBASE database was used to assign motifs to restriction modification systems [57]. A self-written Perl script was used to search the genome of *A. veronii* C4 for the positions of all AveC4I motifs (https://github.com/JiayueMa0529/perl_findMotif/blob/master/1_motif_loc.pl). The genomic distribution of the recognition sequence was visualized using the Gene-Density tool available in Hiplot [58]. The genes harbouring the recognition sequence were then annotated in their corresponding KEGG pathways. Enrichment analysis was employed using OmicShare tools (https://www.omicshare.com/tools/Home/Soft/pathwaygseasenior), and the outcomes were visualized through ImageGP [59].

## Results and discussion

### *A. veronii* has an ‘open’ pan-genome

**Fig. 1.**
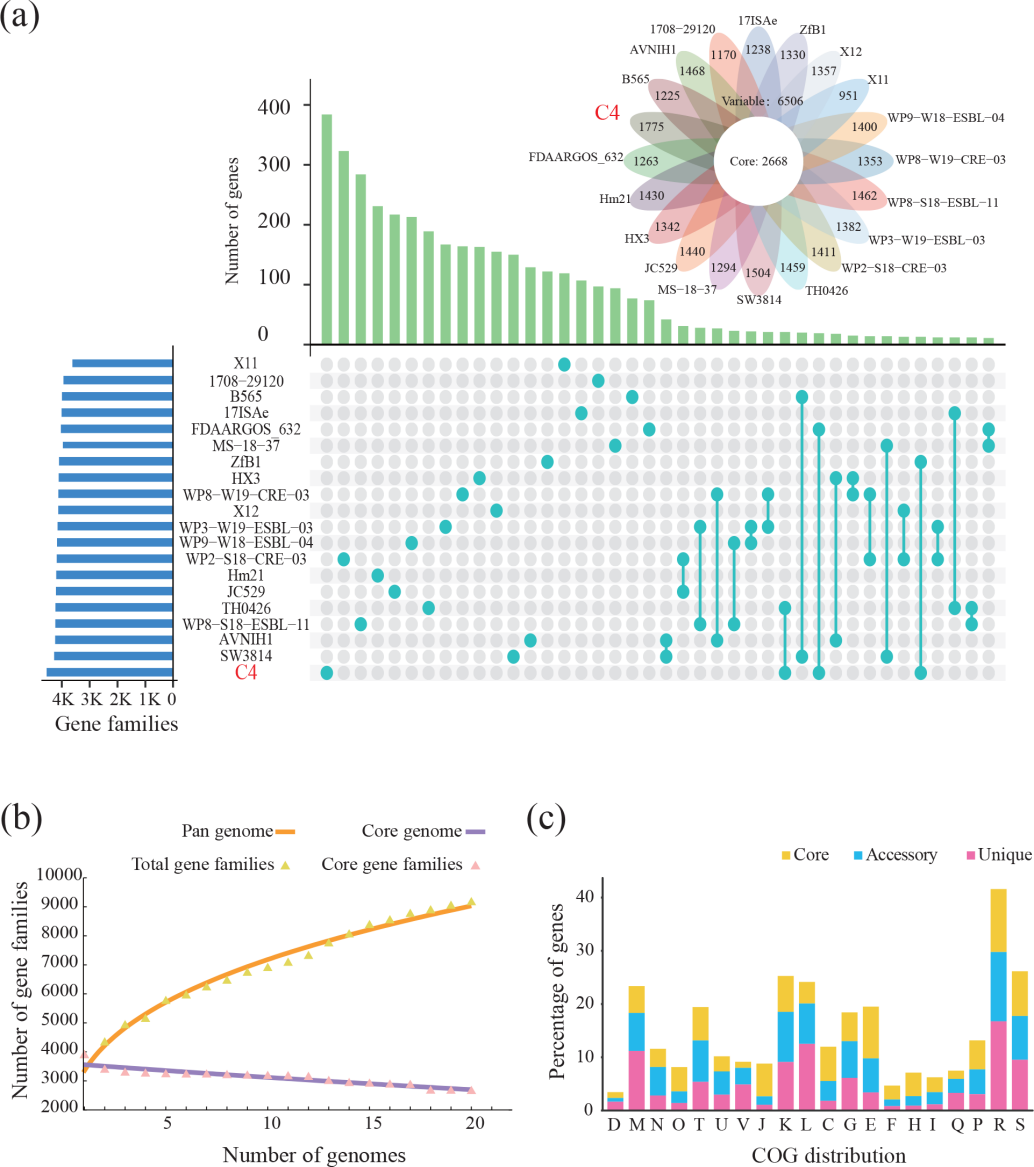
Pan-genome structure of 20 *Aeromonas veronii* strains. (**a**) Comparative genomic map of 20 *A. veronii* strains. The blue bar represents the number of gene families in each strain. The green bar on the genomic map denotes the count of either unique genes (glaucous dots) present in individual strains, or accessory genes (glaucous dots connected by the glaucous line) found in at least two strains. The flower plot represents the number of core genes and the remaining genes (defined as variable genes) in each strain. (**b**) Gene accumulation curves for the pan-genome and core genome of 20 *A. veronii* strains. (**c**) Distribution of Clusters of Orthologous Groups (COG) functional annotations of 20 *A. veronii* strains. [C] Energy production and conversion, [D] Cell cycle control, cell division, chromosome partitioning, [E] Amino acid transport and metabolism, [F] Nucleotide transport and metabolism, [G] Carbohydrate transport and metabolism, [H] Coenzyme transport and metabolism, [I] Lipid transport and metabolism, [J] Translation, ribosomal structure and biogenesis, [K] Transcription, [L] Replication, recombination and repair, [M] Cell wall/membrane/envelope biogenesis, [N] Cell motility, [O] Posttranslational modification, protein turnover, chaperones, [P] Inorganic ion transport and metabolism, [Q] Secondary metabolite biosynthesis, transport and catabolism, [R] General function prediction only, [S] Function unknown, [T] Signal transduction mechanisms, [U] Intracellular trafficking, secretion and vesicular transport, [V] Defence mechanisms, [Y] Nuclear structure.

The genome of *A. veronii* C4 was sequenced, which was found to have a G+C content of 58.50 % and a total length of 4 926 096 bp. This strain harboured 4724 protein-coding genes and 168 noncoding RNA genes. After downloading the genomes of the other 19 *A *. *veronii* strains, whose assembly levels were complete, from the National Center for Biotechnology Information (NCBI) Genome database, their pan-genome was acquired, and this contained 9174 orthologous gene families (Table S1, available in the online version of this article). Of these, 2668 genes were shared by the 20 strains (defined as core genes), 3459 were unique to individual strains (defined as unique genes), and the remaining were accessory genes, indicating a high degree of genetic variation (Fig. 1a and Table S1). The number of unique genes in different *A. veronii* strains exhibited significant variation, ranging from 74 to 384 (Fig. 1a and Table S2). After removing the restriction of assembly level, the genomes of 233 *A *. *veronii* strains including *A. veronii* C4 were obtained and the number of unique and accessory genes increased accordingly (Table S3). There were 2179 core genes for the 233 strains (Table S3). This suggested that certain strains may accept essential foreign genes and, in some cases, even discard defence mechanisms such as CRISPR (clustered regularly interspaced short palindromic repeats) in order to facilitate gene integration [13, 60, 61]. Notably, *A. veronii* C4 had a relatively large genome among the compared strains, implying that it had a greater capacity for environmental adaptability and had probably acquired genes through HGT.

The gene accumulation curves depicted in Fig. 1(b) showed that the pan-genome of 20 *A *. *veronii* strains was ‘open’, as its size continued to increase without showing signs of saturation with the inclusion of more genomes. In contrast, the size of the core genome gradually decreased but did not seem to approach a plateau. To ensure the robustness of the results, the pan-genome of 233 *A *. *veronii* strains was also analysed and the same phenomenon was still observed (Fig. S1A). This indicated that *A. veronii* strains frequently and readily incorporated genes from other species, resulting in a constant increase in the diversity and functionality of genes. Interestingly, bacteria with ‘closed’ pan-genomes are typically found in stable environments, such as the commensal bacterium *Staphylococcus lugdunensis* [62]. However, most free-living bacteria possess ‘open’ pan-genomes to adapt to variable environments. In fact, nine bacteria have been shown to have ‘open’ pan-genomes [63], and even the bacterial domain as a whole does [64].

An ‘open’ pan-genome confers a greater adaptive potential for a species to thrive in varying ecological niches. To unravel the functional characteristics of the pan-genome of the 20 *A *. *veronii* strains, we conducted functional annotation of the core, unique and accessory genes using the COG database (Fig. 1c). A substantial portion of the pan-genome, representing 67.76 %, was not well characterized with respect to functional annotation, as indicated by ‘general function prediction only’ or ‘function unknown’. The core genome exhibited diverse functions and played a crucial role in various biological processes, such as ‘amino acid transport and metabolism’ (9.73 %), ‘transcription’ (6.74 %), ‘energy production and conversion’ (6.45 %), ‘signal transduction mechanisms’ (6.26 %), and ‘translation ribosomal structure and biogenesis’ (6.15 %) (Fig. 1c and Table S3). The accessory genome was primarily involved in ‘transcription’ (9.41 %), while the unique genome was enriched for genes related to ‘replication, recombination and repair’ (12.52 %) and ‘cell wall/membrane/envelope biogenesis’ (11.19 %) (Fig. 1c and Table S4). When analysing the pan-genome of 233 *A *. *veronii* strains, the functional repertoire of three categories of genes did not show a clear change (Fig. S1B and Table S5).

### Analysis of gene gain and loss in the evolution of *A. veronii*

**Fig. 2.**
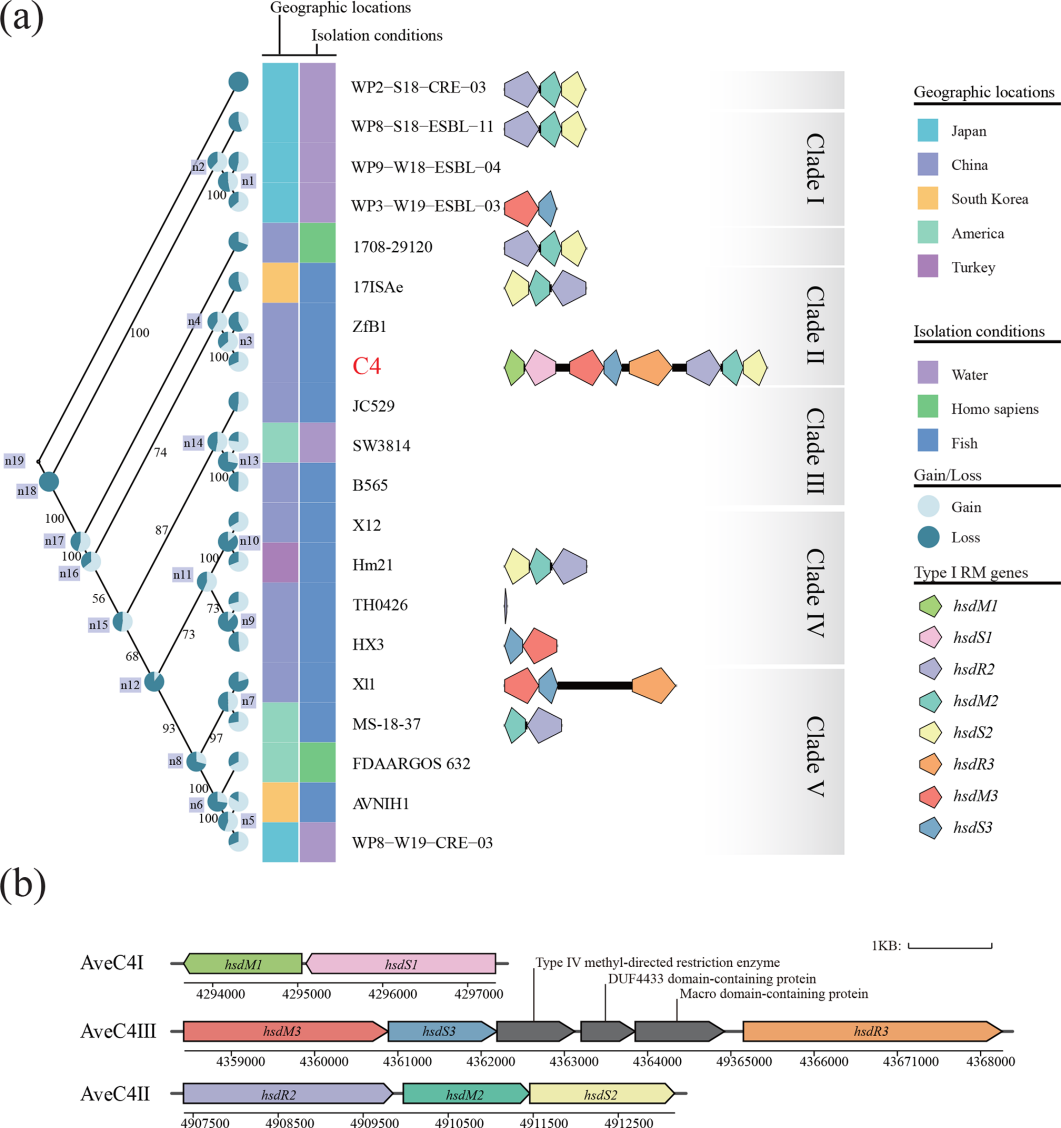
Distribution of *hsd* genes in 20 *Aeromonas veronii* strains. (**a**) Phylogenetic analysis of 20 *A *. *veronii* strains. The phylogenetic tree based on 2728 single-copy core genes of the 20 strains is divided into five clades. Gene gain (light blue) and loss (dark blue) are represented by pie charts. Geographical location and isolation conditions are represented by heat maps. The gene cluster consists of homologous genes of eight *hsd* genes in *A. veronii* C4. Bootstrap values from 1000 replicates are shown. (**b**) Gene clusters of AveC4I, AveC4II and AveC4III in *A. veronii* C4.

To analyse the dynamics of gene families in the evolution of *A. veronii *, we reconstructed a phylogenetic tree of 20 strains based on 2728 single-copy core genes (Fig. 2a). Based on the topological structure and evolutionary distance, the tree was divided into five clades, with the exception of the independently evolved strains 1708-29120 and WP2-S18-CRE-03 [65]. The strains in Clade I, including WP8-S18-ESBL-11, WP9-W18-ESBL-04 and WP3-W19-ESBL-03, were isolated from water in Tokyo, Japan. Interestingly, strain WP8-W19-CRE-03 was also isolated from the same location but belonged to Clade V (Fig. 2a). Similarly, strains X11 and X12 exhibited distinct core genomes, despite being isolated from the same location. Strains C4 and ZfB1 evolved from a common ancestor and together with strain 17ISAe constituted Clade II (Fig. 2a).

The orthologous genes of ancestors was inferred (Table S6), and the ancestral genome of the 20 *A *. *veronii* strains was estimated to contain 4152 gene families (Table S7), while the ancestral genome of 29 *Aeromonas* species contained 3821 gene families [66]. The phylogenetic tree of 20 *A *. *veronii* strains showed evidence of gene gain and loss, indicating a dynamic evolutionary process in *A. veronii* (Fig. 2a and Table S7). Gene gain was more frequent than gene loss in most strains (Fig. 2a and Table S7), suggesting that *A. veronii* acquired new genes through HGT or other mechanisms to adapt to diverse lifestyles [66]. In contrast, gene loss was more common in the 29 species of *Aeromonas *, reflecting vertical descent in the species [65]. The patterns of gene gain and loss were not consistent across *A. veronii* strains, even among closely related strains (Fig. 2a). For example, strain C4 gained 245 genes and lost 114 genes, while strain ZfB1 gained 144 genes and lost 196 genes. These results suggested that gene gain and loss had played a major role in driving the evolution and shaping the genetic diversity of *A. veronii *.

### Scattered distribution of *hsd* genes in *A. veronii*

Three *hsdM* genes (Locus tags: ONR73_20345, ONR73_23265 and ONR73_20695), three *hsdS* genes (Locus tags: ONR73_20340, ONR73_23270 and ONR73_20700) and two *hsdR* genes (Locus tags: ONR73_20720 and ONR73_23260) were found. These *hsd* genes constituted one Type I MTase and two Type I RM systems (Fig. 2a). They were all located on defence islands and were designated as AveC4I, AveC4II and AveC4III [67]. AveC4I was composed of *hsdM1* (Locus tag: ONR73_20345) and *hsdS1* (Locus tag: ONR73_20340), while AveC4II was encoded by *hsdR2* (Locus tag: ONR73_23260), *hsdM2* (Locus tag: ONR73_23265) and *hsdS2* (Locus tag: ONR73_23270). AveC4III consisted of *hsdR3* (Locus tag: ONR73_20720), *hsdM3* (Locus tag: ONR73_20695) and *hsdS3* (Locus tag: ONR73_20700). In AveC4I and AveC4III, the *hsdM* and *hsdS* genes were closely located, but *hsdR* was either absent or separated, resulting in the decay of an RM cluster or the potential for conjunct function with other RM systems [68]. Between *hsdS3* and *hsdR3*, three genes were integrated, including a Type IV methyl-directed restriction enzyme, a DUF4433 domain-containing protein and a Macro domain-containing protein (Fig. 2b). These mosaic genes probably arose from evolutionary recombination and may be redundant, gradually fusing or disappearing in the near future, ultimately leading to the development of a tightly continuous Type I RM system [69].

Comparative genomic analysis was conducted to examine homologous genes of the Type I RM systems and MTase in 19 other *A. veronii* strains, in comparison with *A. veronii* C4 (Fig. 2a). Homologous genes of AveC4II or AveC4III were present in ten strains, while the remaining strains lacked these genes. Notably, AveC4I only existed in *A. veronii* C4 when analysing 233 *A *. *veronii* strains (Table S8). The Type I RM systems and MTase play an important role in enhancing the environmentally adaptive capacity of bacterial strains. Under high adverse pressure, strains tend to acquire Type I RM systems and MTase from outside sources and regulate gene expression to adapt by altering specific motifs. Since the Type I RM system requires ATP consumption, strains tend to discard the system to reduce metabolic burden when the stress is relieved [60, 61, 70–72]. Despite this, AveC4I, AveC4II and AveC4III have been retained in the genome of *A. veronii* C4, probably due to experiencing tremendous pressure during the course of evolution.

### Incongruence between the phylogenetic trees hinted at potential HGT of AveC4I

**Fig. 3.**
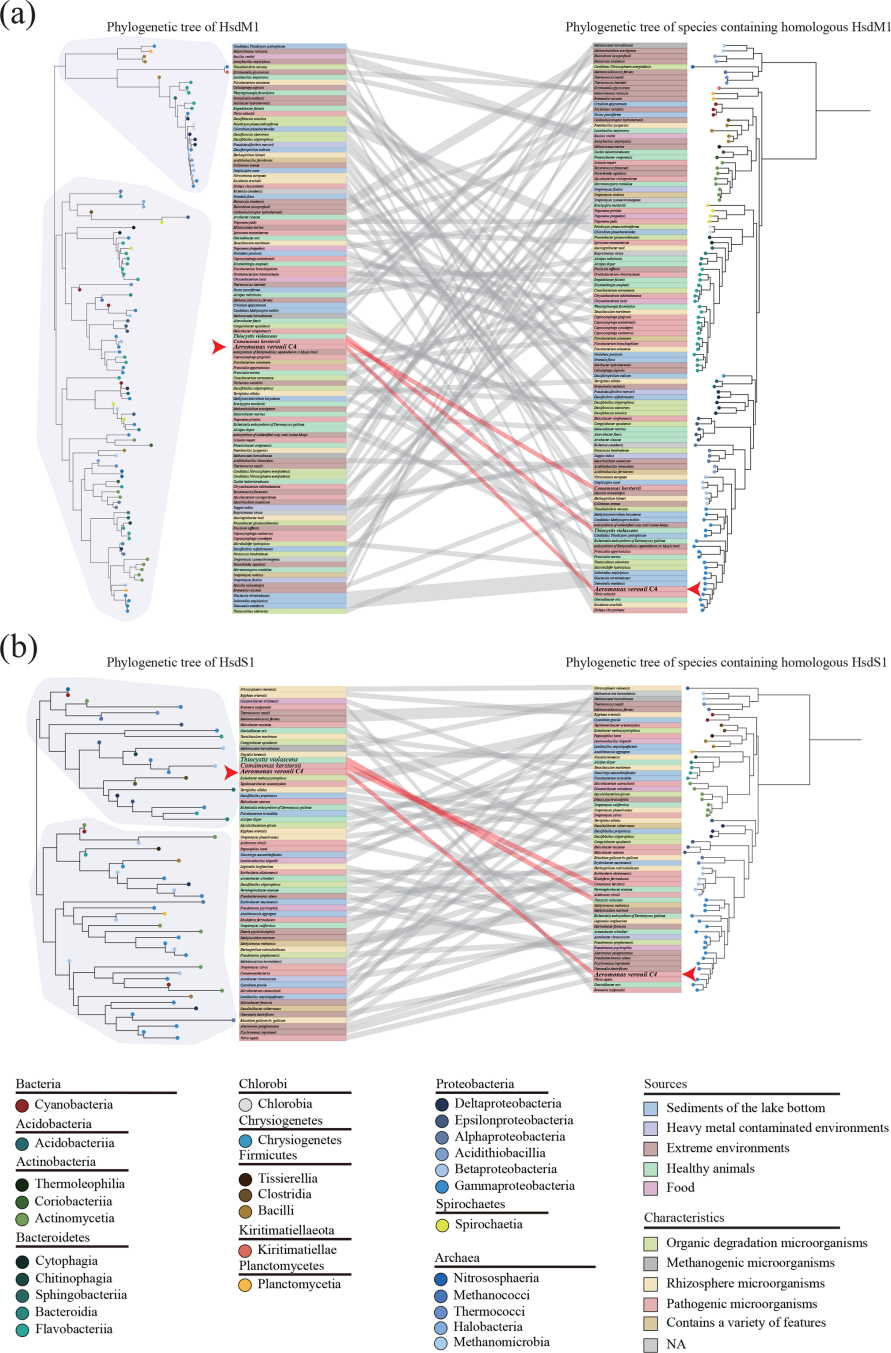
Comparison between phylogenetic trees. (**a**) The phylogenetic tree of HsdM1 was reconstructed based on 109 homologous HsdM1 (left), and the phylogenetic tree of the 107 species containing homologous HsdM1 was reconstructed based on 21 single-copy core genes (right). (**b**) The phylogenetic tree of HsdS1 was reconstructed based on 59 homologous HsdS1 (left), and the phylogenetic tree of the 58 species containing homologous HsdS1 was reconstructed based on 26 single-copy core genes (right). In (a) and (b) dots of various colours symbolize the species belonging to different classes. Different background colours highlight the species that come from diverse sources or have different characteristics. Lines connect the same species. *A. veronii* C4 is marked with a red arrowhead.

We analysed 3357 bacterial and 220 archaeal reference genomes that were fully assembled in order to identify homologous proteins of HsdM1 and HsdS1. Our analysis identified 109 homologous HsdM1 in 107 species (Fig. 3a), whereas only 59 homologous HsdS1 were identified in 58 species (Fig. 3b), probably due to the highly variable TRDs on both ends of the S subunit in different species [4]. A total of 152 species, mostly belonging to the classes *Gammaproteobacteria *, *Flavobacteriia *, *Actinomycetia* and *Betaproteobacteria *, contained either HsdM1 or HsdS1. Of these species, 13 contained both HsdM1 and HsdS1. To explore the evolutionary relationships between these 152 species, a phylogenetic tree was reconstructed using 18 single-copy core genes (Fig. S2A, B). Species of archaea from five classes were clustered into a large clade and evolved independently from bacteria. Similarly, species of bacteria from the same class tended to be clustered together. The species of *Gammaproteobacteria* evolved later and shared a common ancestor with the species of *Betaproteobacteria *. *A. veronii* C4, classified in *Gammaproteobacteria *, was clustered together with the genus *Shewanella* (Fig. S2A, B).

Phylogenetic trees of HsdM1 and HsdS1 as well as the phylogenetic trees of the species containing homologous HsdM1 or HsdS1 were reconstructed (Fig. 3a, b). Unlike the phylogenetic tree of the species, the phylogenetic tree of HsdM1 or HsdS1 separated species belonging to the same class into different clades, suggesting that both *hsdM1* and *hsdS1* have undergone HGT. The phylogenetic tree for HsdM1 was divided into two large clades (Fig. 3a), each containing several small clades composed of species from the classes *Flavobacteriia *, *Actinomycetia* and *Gammaproteobacteria *. The phylogenetic tree for HsdS1 was also divided into two clades (Fig. 3b), but the distribution of species was more irregular. Even for two species in the same clade, the total branch length was high, further explaining the high variability of HsdS1. In the phylogenetic trees of HsdM1 and HsdS1, *A. veronii* C4 was most closely related to *Comamonas kerstersii* (Fig. 3a, b). Between the two species, the protein sequences of HsdM1 and HsdS1 shared 88 and 60% identity, respectively. *Comamonas kerstersii *, like *A. veronii* C4, is a drug-resistant pathogen that causes peritoneal infections, bacteraemia and other diseases [73, 74].

### AveC4I has complex HGT routes

Using RANGER-DTL, HGT and gene duplication events were inferred to reconstruct the evolutionary history of AveC4I. The analysis revealed extensive HGTs of *hsdM1* and *hsdS1*, which were not limited to species within the same class but also occurred between bacteria and archaea (Fig. S2). Although several species possessed *hsdM1* or *hsdS1*, only the *hsdM1* of *Candidatus* Nitrososphaera evergladensi was found to be duplicated (Fig. S2A).

The evolutionary history of *hsdM1* was revealed to involve extensive HGT events, as inferred by RANGER-DTL (Fig. S2). HGT of *hsdM1* was not limited to species within the same class, but also occurred between species of different classes, including bacteria and archaea. In total, 29 species and ten putative ancestors were predicted as donors of *hsdM1*, including three species of archaea (* Methanocaldococcus fervens* of *Methanococci *, *Methanosaeta harundinacea* of *Methanomicrobia* and *Thermococcus nautili* of *Thermococci *) (Fig. S2A). The *hsdM1* of these three species was transferred to the classes *Bacteroidia *, *Epsilonproteobacteria *, *Gammaproteobacteria* and *Acidithiobacillia *. Among these donor archaea, two strains were isolated from extreme environments, and only one of them transferred *hsdM1* to the bacteria in the same environment. The remaining recipient bacteria were isolated from lake bottom sediments or healthy animals. The phylogenetic tree of HsdM1 showed several clades within *Flavobacteriia *, but their topological structures were inconsistent with those in the phylogenetic tree of the species (Fig. 3a). Based on the prediction of HGT routes, we speculated that HGT of *hsdM1* occurred within the class *Flavobacteriia *. Eight routes were obtained, and two of the donor species were isolated from extreme environments, while the majority of recipients displayed pathogenicity (Fig. S2A and Table 1). In *Cyanobacteria *, HGT of *hsdM1* did not occur, suggesting that the *hsdM1* of these species was acquired through vertical gene transfer. Although numerous species of *Gammaproteobacteria* possessed *hsdM1*, there were relatively few HGT events within the class, and the majority of *hsdM1* were derived from ancestors (Fig. S2A).

**Table 1.** Horizontal gene transfer of *hsdM1* within *Flavobacteriia
*

Donor	Source/characteristic	Recipient(s)	Source/characteristic
* Cellulophaga algicola *	Extreme environment	* Flavobacterium anhuiense * * Wenyingzhuangia fucanilytica *	Rhizosphere microorganism Healthy animal
* Chryseobacterium lactis *	Food	* Ornithobacterium rhinotracheale *	Pathogenic microorganism
* Elizabethkingia anophelis *	Pathogenic microorganism	*Capnocytophaga endodontalis*	Pathogenic microorganism
* Flavobacterium columnare *	Contains a variety of features	* Capnocytophaga gingivalis *	Pathogenic microorganism
*Maribacter hydrothermalis*	Extreme environment	* Empedobacter falsenii *	Healthy animal
n38	–	* Flavobacterium branchiophilum *	Pathogenic microorganism
n28	–	n37	–

In contrast to *hsdM1*, the horizontal transfer of *hsdS1* was typically observed in a radial pattern, especially in *Proteobacteria* (Fig. S2B). The transfer of *hsdS1* from *Helicobacter cetorum* (* Epsilonproteobacteria *) to *Terriglobus albidus* (* Acidobacteriia *) and *Desulfobulbus propionicus* (* Deltaproteobacteria *) resulted in the initial appearance of *hsdS1* in these species. *Pseudomonas qingdaonensis* and *Azotobacter chroococcum* in the same clade frequently transferred *hsdS1* to other species, including four species of *Actinomycetia *. Species of *Cyanobacteria* acquired *hsdS1* from other classes, but did not transfer it to other species. The donor strains were pathogens or microbes with organic degradation ability. The recipient bacteria mostly were pathogens. Interestingly, *A. veronii* C4 received *hsdM1* from *Comamonas kerstersii* (Fig. S2A) and donated *hsdS1* to *Comamonas kerstersii* (Fig. S2B), resulting in a win–win situation. They were all isolated from China and were pathogenic bacteria, indicating that there may be genetic exchange among pathogenic bacteria in the same region.

### AveC4I is associated with the chemotaxis of *A. veronii* C4

Seven methylated motifs, including ‘GTANNNNNNCTTC’, ‘GAAGNNNNNNTAC’, ‘GCCAYNNNNNDTGA’, ‘TCAHNNNNNRTGGC’, ‘CCAYNNNNNCTG’, ‘CAGNNNNNRTGG’ and ‘GATC’, were identified as the recognition motifs when analysing the SMRT sequencing data. To search for homologous proteins and their corresponding recognition motifs, the sequences of HsdS1, HsdS2 and HsdS3 were submitted to the REBASE database (http://tools.neb.com/blast/) [57]. The protein S.Pfe13482III was found to exhibit 11% sequence identity with HsdS1 and to recognize the conserved motif ‘GAAYNNNNNNCTG’. The protein S.SenS922ORF19115P demonstrated 53 % sequence identity with HsdS2 and was observed to specifically recognize the conserved motif ‘GTANNNNNNNNGTTC’. The protein S.Ecos1613ORF5225P exhibited 32 % sequence identity with HsdS3 and was found to recognize the conserved motif ‘CCAYNNNNNGTTY’. Given that TRDs have been shown to recognize analogous sequences with a minimum of 25 % identity in HsdS [4], referring to previous research methods [75], we assigned the sequences ‘GCCAYNNNNNDTGA’, ‘GTANNNNNNNNCTTC’ and ‘CCAYNNNNNCTG’ to AveC4I, AveC4II and AveC4III, respectively.

**Fig. 4.**
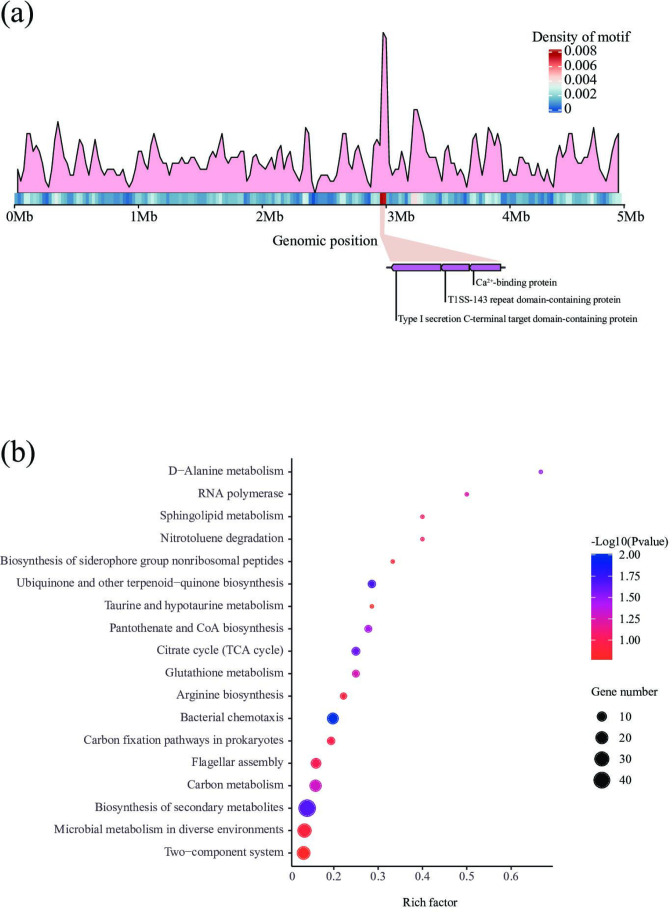
Methylome analysis of AveC4I. (**a**) Distribution of the motif methylated by AveC4I in *A. veronii* C4. The motif is enriched in the region (2.9–3.0 Mb), which includes ONR73_14145 (Type I secretion C-terminal target domain-containing protein), ONR73_14150 (T1SS 143 repeat domain-containing protein) and ONR73_14155 (Ca^2+^-binding protein). (**b**) Scatter plot of KEGG analysis of the genes with the motif. The ‘Bacterial chemotaxis’ pathway showed the most significant enrichment (hypergeometric test, *P*<0.01).

The 14-base novel motif, ‘GCCAYNNNNNDTGA/TCAHNNNNNRTGGC’, was methylated by AveC4I, and included five arbitrary bases in the middle and specific bases at both ends recognized by the TRDs of HsdS1. A total of 571 sequences matching this motif were found dispersed throughout the genome (Fig. 4a), with 509 of these sequences located in 462 genes. Of these genes, 34 contained at least two matched sequences. Notably, three adjacent genes (ONR73_14145, ONR73_14150 and ONR73_14155) spanning from 2974154 to 2983700 bp each contained more than five matched sequences (Fig. 4a), indicating that they were likely to be important regulatory targets of AveC4I. They encode the Type I secretion C-terminal target domain-containing protein, Type I secretion system (T1SS)−143 repeat domain-containing protein, and RTX toxin-related Ca^2+^-binding protein, respectively. Recognition of the Type I secretion C-terminal target domain-containing protein is triggered by the C-terminal secretion signal, which leads to assembly of the functional transenvelope complex and confers specificity to the recognition by the ABC protein and RTX toxins [76]. The T1SS 143 repeat domain-containing protein tends to share several properties with the RtxA protein of *Vibrio cholerae* [77]. The RTX toxin-related Ca^2+^-binding protein can promote the secretion of RTX, which is a type of bacterial exotoxin and can cause damage to host cells [78]. The expression of genes in regions enriched with methylation is regulated by methylation status [22]. DNA methylation can either activate or repress a gene, depending on the presence of other DNA-binding proteins that compete with DNA MTases [79, 80]. The results implied that AveC4I played a role in the regulation of virulence.

KEGG annotation and enrichment analyses were conducted to investigate the functions of the 462 genes containing the novel motif recognized by AveC4I (Fig. 4b). These genes were primarily involved in several pathways, including ‘Carbon metabolism’, ‘Biosynthesis of amino acids’, ‘Bacterial chemotaxis’ and ‘Flagellar assembly’. Among them, the ‘Bacterial chemotaxis’ pathway showed significant enrichment (hypergeometric test, *P*<0.01). A total of 16 genes were annotated in the pathway, including *cheY*, *cheB* and the gene encoding methyl-accepting chemotaxis protein. The expression of these genes related to chemotaxis is suggested to be affected by methylation of AveC4I. Bacterial chemotaxis is a fundamental process that plays a critical role in a multitude of biological processes. It encompasses the directed movement of bacteria towards favourable chemical gradients, while avoiding toxic chemical gradients [81–83]. Depletion of chemotaxis-related genes has been shown to affect the antimicrobial susceptibilities of *Aeromonas hydrophila* [84]. Chemotaxis not only facilitates the ability of *Shewanella oneidensis* MR-1 to colonize but also maintains metabolic activity in regions where the antibiotic ciprofloxacin exhibits high toxicity [85]. The chemotaxis histidine kinase CheA has the ability to phosphorylate not only its specific response regulator CheY2, but also one of the response regulators involved in the pathway that mediates biofilm formation, FlmD [86]. The chemotaxis genes *cheY* and *cheW* in *Helicobacter pylori* also influence its pathogenicity in the stomach [87]. In *Pseudomonas aeruginosa *, *cheB2* assists in adapting to the environment [88]. The observed enrichment of genes within the pathway provided compelling evidence supporting the involvement of AveC4I in the regulation of bacterial chemotaxis and associated biological processes. These findings suggested a potential relationship between AveC4I and drug resistance of *A. veronii *.

## Conclusions

Comparative genomic and phylogenetic analyses have revealed that *A. veronii* possesses an ‘open’ pan-genome, rendering it susceptible to the acquisition of genes from other species via extensive HGTs. Consequently, Type I RM systems and MTase genes were dispersed throughout the *A. veronii* genome. Of all the *A. veronii* strains examined, *A. veronii* C4 possessed a larger genome and greater number of Type I system genes. The AveC4I gene was unique to *A. veronii* C4 and was determined to have been horizontally transferred from *Comamonas kerstersii *. SMRT sequencing and methylation analysis revealed that the methylation sites of AveC4I were clustered on three genes associated with virulence. The expression of genes in regions enriched with methylation is regulated by the methylation status [22]. It is plausible that AveC4I may exert an influence on the virulence of *A. veronii* C4. Pathway analyses of genes featuring AveC4I recognition motifs implied that AveC4I may regulate drug resistance pathway. Methylation has been reported to affect virulence and drug resistance in various bacteria, such as *Streptococcus pyogenes* and *Mycoplasma pneumoniae* [18, 21]. Therefore, we speculate that Type I RM systems also could be a promising therapeutic target for the treatment of diseases related to *A. veronii *.

## Supplementary Data

Supplementary material 1

Supplementary material 2
