# Supplementary material for: Acquisition of Type I methyltransferase via horizontal gene transfer increases the drug resistance of Aeromonas veronii
Source: Microb Genom. 2023 Sep 27;9(9):001107. doi: 10.1099/mgen.0.001107 (PMC10569733; doi:10.1099/mgen.0.001107)
Supplement: Supplementary material 1 [file mgen-9-1107-s001.pdf]

A

B

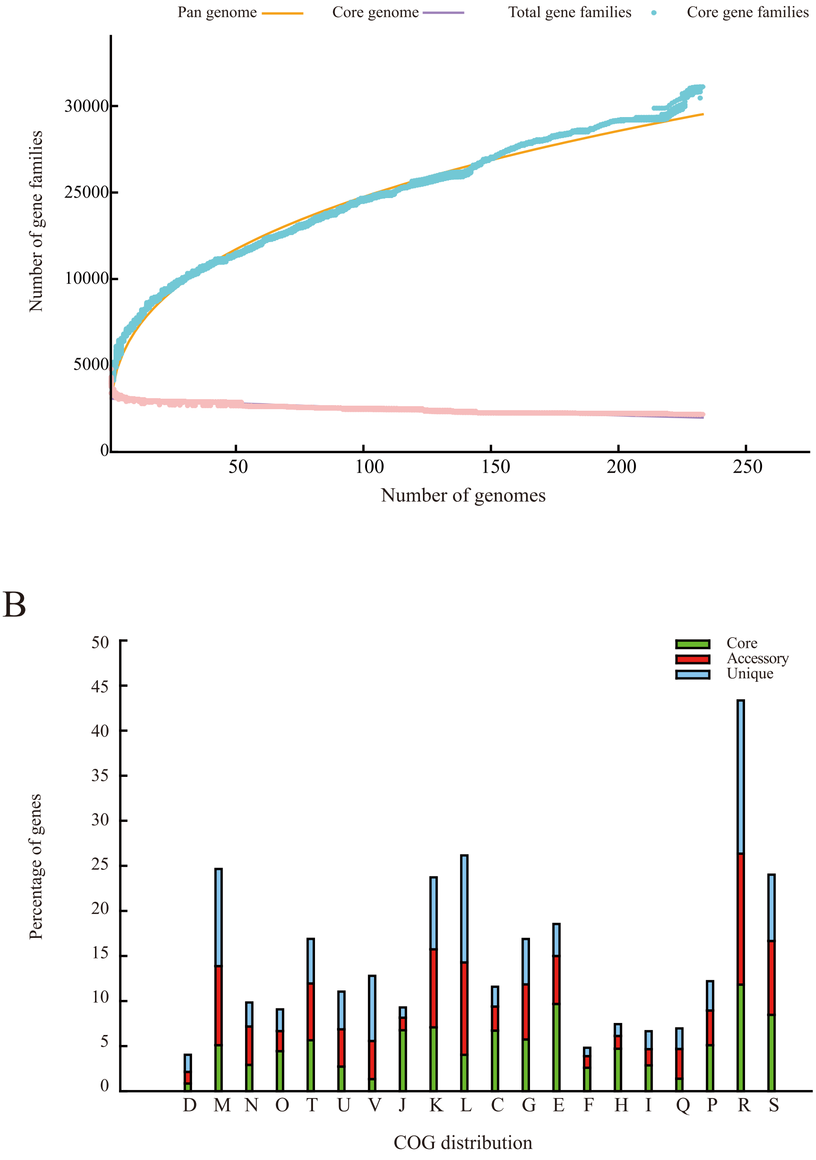

Figure S1. Pan-genome structure of 233 *Aeromonas veronii* strains. (A) Gene accumulation curves for pan-genome and core genome of 233 *A. veronii* strains; (B) Distribution of Clusters of Orthologous Groups (COG) functional annotations of 233 *A. veronii* strains. [C] Energy production and conversion, [D] Cell cycle control, cell division, chromosome partitioning, [E] Amino acid transport and metabolism, [F] Nucleotide transport and metabolism, [G] Carbohydrate transport and metabolism, [H] Coenzyme transport and metabolism, [I] Lipid transport and metabolism, [J] Translation, ribosomal structure and biogenesis, [K] Transcription, [L] Replication, recombination and repair, [M] Cell wall/membrane/envelope biogenesis,[N] Cell motility, [O] Posttranslational modification, protein turnover, chaperones, [P] Inorganic ion transport and metabolism, [Q] Secondary metabolites biosynthesis, transport and catabolism, [R] General function prediction only, [S] Function unknown, [T] Signal transduction mechanisms, [U] Intracellular trafficking, secretion, and vesicular transport, [V] Defense mechanisms, [Y] Nuclear structure.

A

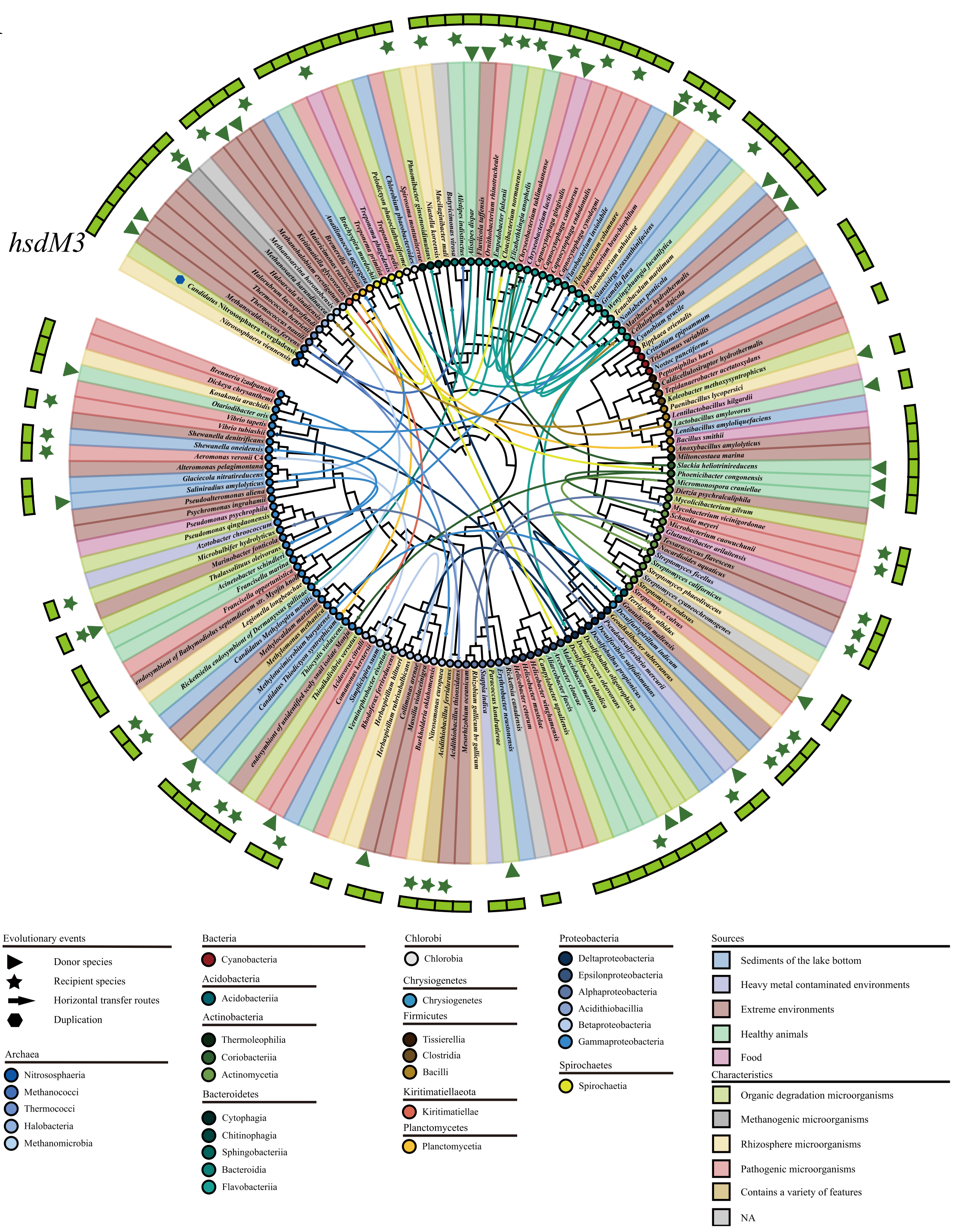

hsdM3
Evolutionary events
Donor species
Recipient species
Horizontal transfer routes
Duplication
Archaea
Nitrososphaeria
Methanococci
Thermococci
Halobacteria
Methanomicrobia
Bacteria
Cyanobacteria
Acidobacteria
Acidobacteriia
Actinobacteria
Thermoleophilia
Coriobacteriia
Actinomycetia
Bacteroidetes
Cytophagia
Chitinophagia
Sphingobacteriia
Bacteroidia
Flavobacteriia
Chlorobi
Chlorobia
Chrysiogenetes
Chrysiogenetes
Firmicutes
Tissierellia
Clostridia
Bacilli
Kiritimatiellaeota
Kiritimatiellae
Planctomycetes
Planctomycetia
Proteobacteria
Deltaproteobacteria
Epsilonproteobacteria
Alphaproteobacteria
Acidithiobacillia
Betaproteobacteria
Gammaproteobacteria
Spirochaetes
Spirochaetia
Sources
Sediments of the lake bottom
Heavy metal contaminated environments
Extreme environments
Healthy animals
Food
Characteristics
Organic degradation microorganisms
Methanogenic microorganisms
Rhizosphere microorganisms
Pathogenic microorganisms
Contains a variety of features
NA

B

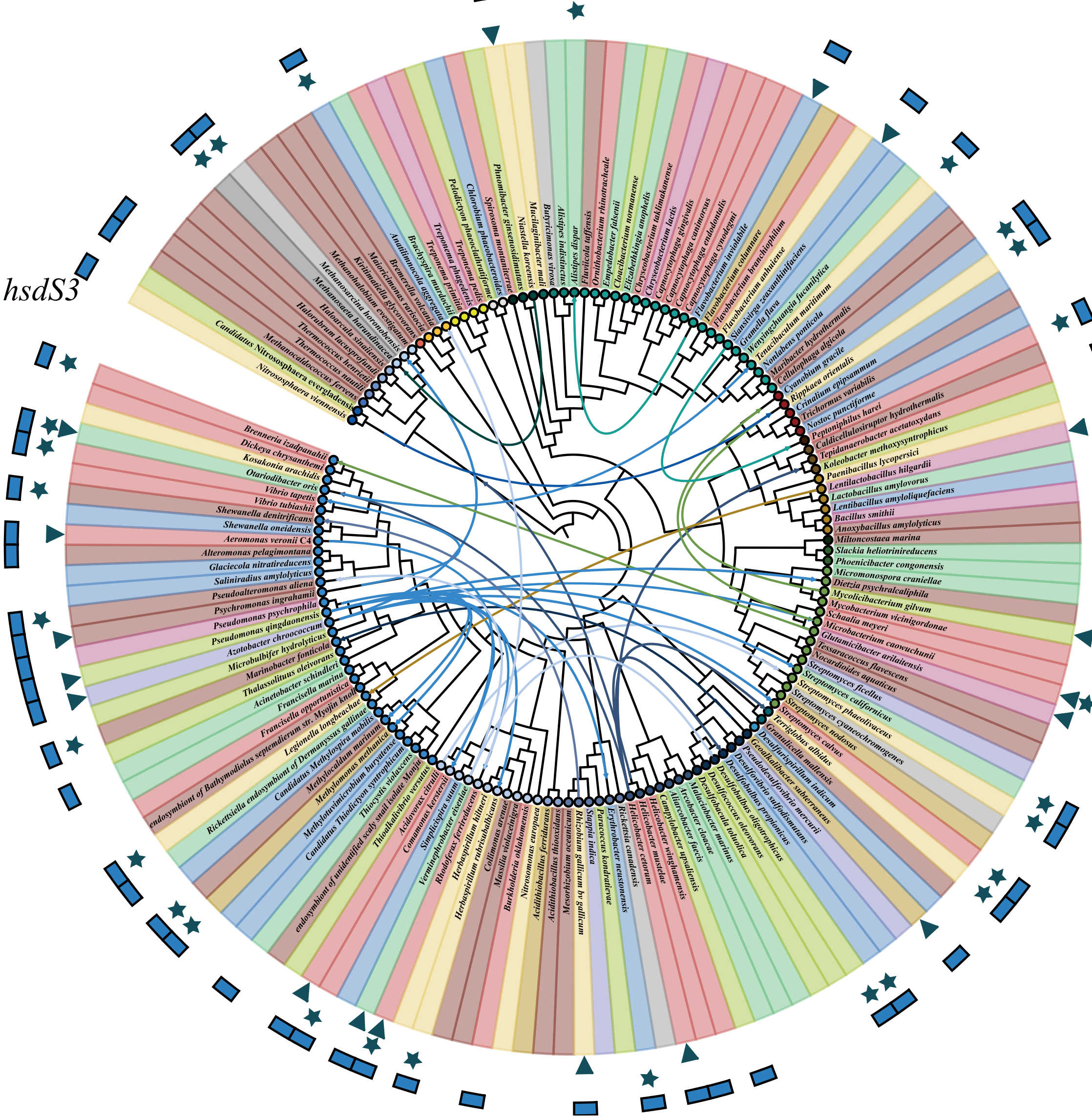

Figure S2. Horizontal gene transfer (HGT) routes of *hsdM1* and *hsdS1*. (A and B) The mid-point-rooted phylogenetic tree was constructed based on 18 single-copy core genes from 152 species containing *hsdM1* or *hsdS1*. If a species contains (A) *hsdM1* or (B) *hsdS1*, it is marked with (A) green or (B) blue box in the outermost circle. The 152 species are highlighted in different background colors based on their source or characteristics and are represented by dots of different colors according to their classifications at the class level. The HGT route of (A) *hsdM1* or (B) *hsdS1* is represented by the curved arrow, which is colored based on the donor species (marked with the triangle). The thickness of the curved arrow indicates the probability of HGT, and the HGT route is shown only when the probability is more than 50%.
